# The miR-17 Family Links p63 Protein to MAPK Signaling to Promote the Onset of Human Keratinocyte Differentiation

**DOI:** 10.1371/journal.pone.0045761

**Published:** 2012-09-24

**Authors:** Ning Wu, Eric Sulpice, Patricia Obeid, Sami Benzina, Frédérique Kermarrec, Stéphanie Combe, Xavier Gidrol

**Affiliations:** 1 CEA, Laboratoire de Biologie à Grande Echelle, Grenoble, France; 2 INSERM, U1038, Grenoble, France; 3 Université Joseph Fourier, Grenoble, France; IPMC, CNRS UMR 7275 UNS, France

## Abstract

The p63 protein plays a key role in regulating human keratinocyte proliferation and differentiation. Although some p63-regulating microRNAs (miRNAs) have been identified in the control of epidermal homeostasis, little is known about miRNAs acting downstream of p63. In this paper, we characterized multiple p63-regulated miRNAs (miR-17, miR-20b, miR-30a, miR-106a, miR-143 and miR-455-3p) and elucidated their roles in the onset of keratinocyte differentiation. We identified RB, p21 and multiple MAPKs as targets of these p63-controlled miRNAs. Upon inhibition of most of these miRNAs, we observed defects in commitment to differentiation that could be reversed by siRNA-mediated silencing of their targets. Furthermore, knockdown of MAPK8 and MAPK9 efficiently restored expression of the early differentiation markers keratin 1 and keratin 10 in p63-silenced primary human keratinocytes. These results highlight new mechanistic roles of multiple miRNAs, particularly the miR-17 family (miR-17, miR-20b and miR-106a), as regulatory intermediates for coordinating p63 with MAPK signaling in the commitment of human mature keratinocytes to early differentiation.

## Introduction

Through intensive studies in the past decades, the understanding of epidermal morphogenesis and the control of its homeostasis has been greatly extended [1]–[3]. When keratinocytes commit to differentiation, they detach from the basal layer and migrate outward into the spinous layer. This process is accompanied by the expression of keratin 1 (K1) and keratin 10 (K10). These two cytokeratins are markers of keratinocyte early differentiation and the basal-spinous switch in the epidermis. Among the molecular actors that control epidermal homeostasis, p63 appears to be a major one. Its roles in the maintenance of proliferative potential of epithelial stem cells, epithelial lineage commitment, differentiation of keratinocytes, and epithelial cell adhesion and survival have been well established [4]–[7].

MicroRNAs (miRNAs) are small non-coding RNAs (normally 18–25 nt) that are widely expressed in plants and animals [8]–[10]. miRNAs repress gene expression at the post-transcriptional level by base pairing within the 3′ untranslated region (3′-UTR) of the target mRNAs [11], [12]. The discovery of miRNAs has added a new dimension to the regulation of gene expression, and every day, more evidence demonstrates their importance in animal development and physiology [13]–[15]. Recent studies have shown that on a global scale, miRNAs can promote differentiation, and their lower expression in tumors could reflect a de-differentiation process [16]. In agreement with this hypothesis, mouse embryonic stem cells lacking dicer fail to differentiate normally [17].

Because p63 is a key regulator of keratinocyte differentiation, we aimed to identify p63-regulated miRNAs in human keratinocytes. A set of miRNAs has been demonstrated to be important in the morphogenesis of skin [18], [19]. Among them, miR-203 has been shown to promote epidermal differentiation *in vivo* and keratinocyte differentiation *in vitro* by restricting proliferative potential and inducing cell cycle exit through one of its important targets, p63 [20]–[22]. In addition to miR-203, miR-302 and miR-92 have recently been reported to repress p63 expression in other tissues [23], [24]. Additionally, it was recently reported that the miR-34 family was under the control of p63 in human keratinocytes and controlled epidermal cell proliferation [25]. However, an exhaustive characterization of miRNAs regulated by p63 is lacking. In this paper, we identified miRNAs that were modulated in p63-depleted human keratinocytes. With specific inhibitors, we found that the commitment to differentiation was significantly reduced upon silencing of a subset of these miRNAs. Among the predicted potential targets of these miRNAs, we identified several mitogen-associated protein kinases (MAPKs) and further evaluated their roles in the onset of human mature keratinocyte differentiation.

## Results

### Characterization of p63-regulated miRNAs

**Figure 1:**
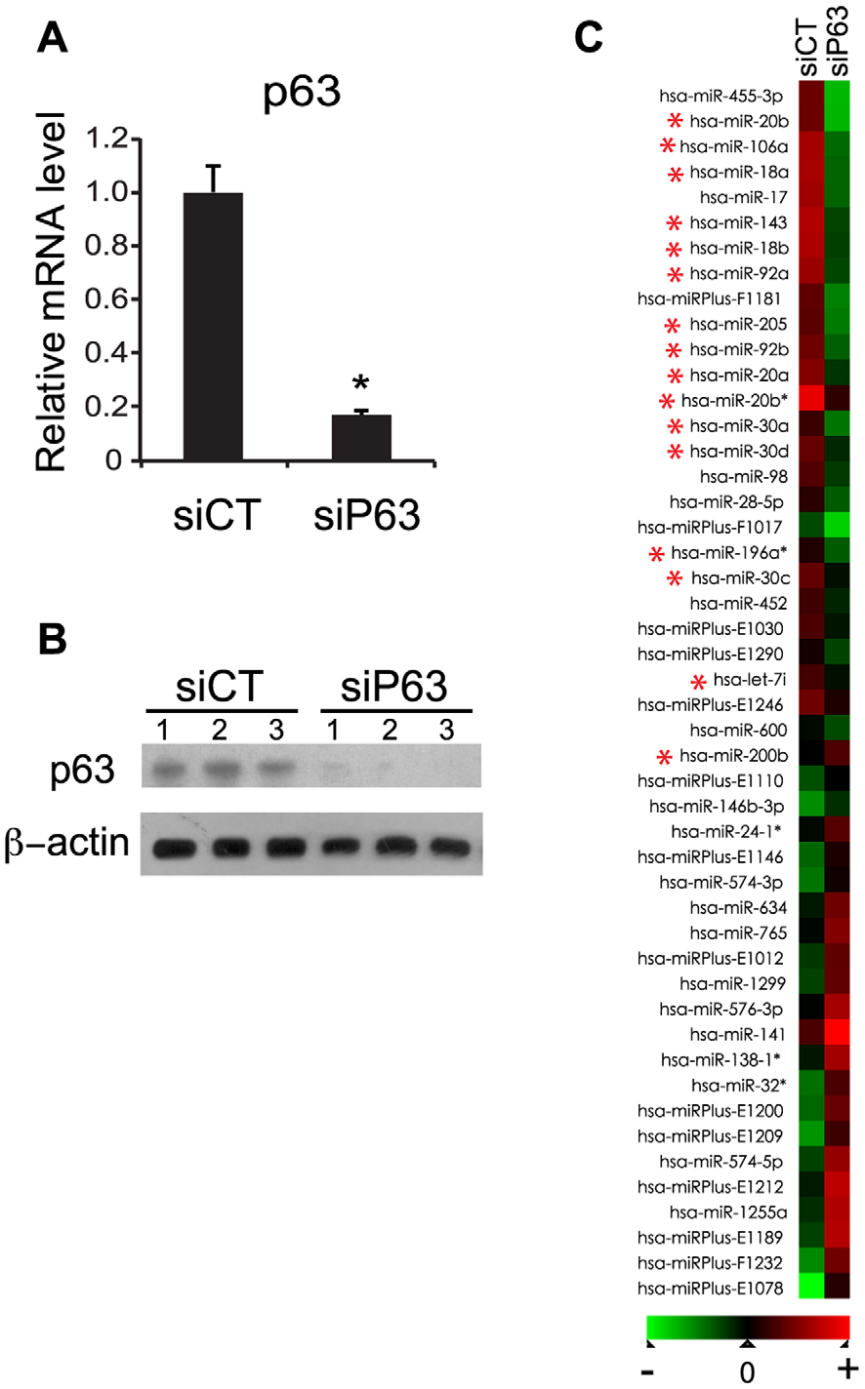
miRNA profiling of p63-depleted HaCaT cells. siRNA-mediated knockdown of p63 was assessed at the mRNA level (**A**) and protein level (**B**) 48 hours post-transfection. The total miRNA extracted (three independent siP63 transfection and three independent miRNA extraction) was processed for miRNA profiling. Error bars represent the s.d. of triplicate experiments. *, p<0.05. (**C**) Heat-map of differentially expressed miRNAs in p63-knockdown keratinocytes compared with control siRNA-treated cells. Red indicates upregulated miRNAs, and green represents downregulated miRNAs. The actual ratio can be found in Table S1. The miRNAs reported by Yi R. et al. are indicated with red stars.

To identify the miRNAs acting downstream of p63, we analyzed miRNA expression profiles in the human keratinocyte HaCaT cell line transfected with siRNA targeting all of the known p63 isoforms and observed a significant silencing of p63 expression at both the mRNA (Figure 1A) and protein levels (Figure 1B). Because human primary keratinocytes exhibit high variability due to both individual variation from donor to donor and the origin of keratinocytes, we used HaCaT cells and three independent biological replicates (independent transfection and independent RNA extraction) to increase reproducibility in miRNA expression profiling. At 48 hours post-transfection, total RNA was extracted and analyzed using the Exiqon microarray platform. We established a heat map of miRNAs that were modulated in p63-knockdown keratinocytes compared with cells transfected with a negative-control siRNA (p<1×10^−3^; Figure 1C) and the actual ratio are given in Table S1. The majority of miRNA microarray results were validated with RT-qPCR (data not shown). Many of these miRNAs (indicated with red stars) were also reported to be differentially expressed in the epidermis and hair follicles of mice [18]. Strikingly, most of the annotated miRNAs were down regulated upon p63 silencing, which is consistent with the view that globally miRNAs would promote differentiation.

### p63-regulated miRNAs Control the Onset of Keratinocyte Differentiation

**Figure 2:**
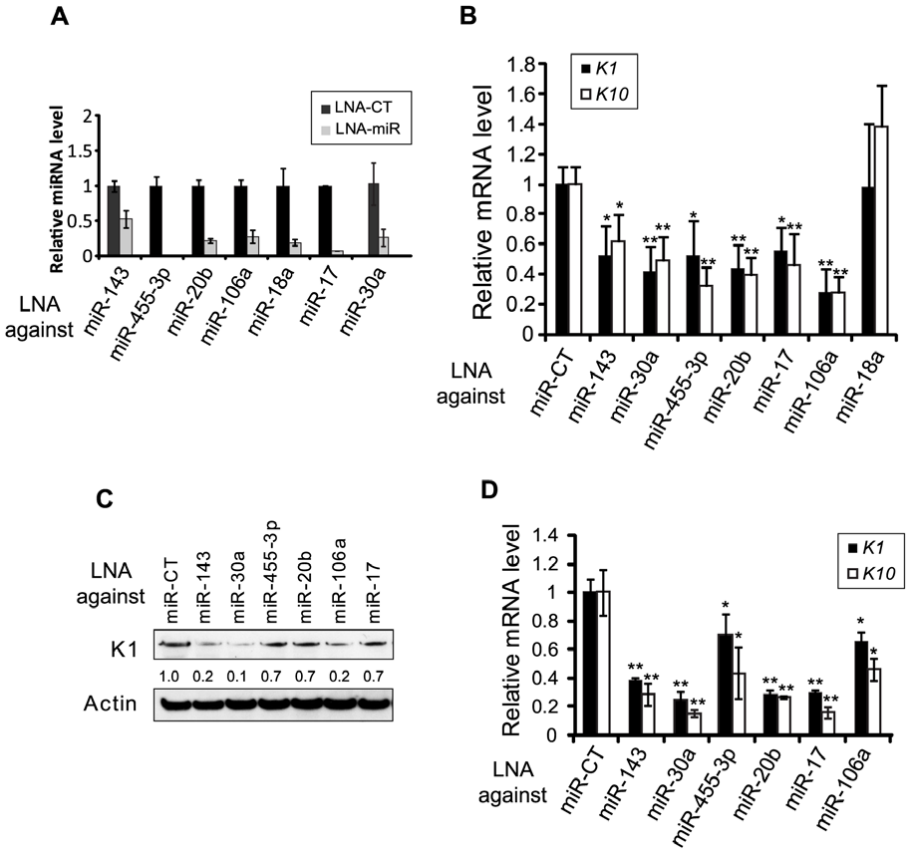
p63-regulated miRNAs involved in the commitment of keratinocyte differentiation. (**A**) LNA-mediated miRNA silencing in HaCaT cells. Relative miRNA expression levels were quantified by RT-qPCR and normalized to U6 snRNA. Error bars represent the s.d. of triplicates. (**B**) The expression of the early differentiation markers *K1* and *K10* after specific miRNAs were silenced was quantified by RT-qPCR. Error bars represent the s.e.m of triplicate experiments. The *t*-test was used for statistical analysis (*, p<0.05; **, p<0.01). (**C**) Immunoblot of K1 in LNA-treated cells. K1 expression levels were quantified and normalized to the loading control β-actin. (**D**) The expression of the early differentiation markers *K1* and *K10* in primary human keratinocytes after specific miRNAs were knocked down was quantified by RT-qPCR. Error bars indicate the s.d. of triplicates.

Because p63 is a well-known regulator of keratinocyte differentiation, we determined whether the p63-regulated miRNAs that we identified were also involved in the regulation of the onset of keratinocyte differentiation. Based on their level of expression in human primary keratinocytes in culture (data not shown) and their biological relevance, we chose several potential candidates from our list: miR-17, miR-18a, miR-20b, miR-30a, miR-106a, miR-143 and miR-455-3p. To investigate the roles of these miRNAs in the onset of differentiation, we knocked down each miRNA using specific miRNA inhibitors, locked nucleic acids (LNA), from Exiqon [26]. We verified the efficiency of the inhibition of each miRNA with RT-qPCR 72 hours after LNA transfection (Figure 2A). Upon the inhibition of each miRNA tested, except miR-18a, we observed the decreased expression of *K1* and *K10* at the mRNA level (Figure 2B) and K1 at the protein level (Figure 2C). Because of our focus on the onset of differentiation we deliberately choose to analyze these two early markers rather than late differentiation markers, such as involucrin, loricrin, filaggrin, etc. Similar experiments were performed in primary human keratinocytes (PHK) (Figure 2D), and the results confirmed those obtained in HaCaT cells. The down-regulation of K1 and K10 upon miRNA silencing suggested that these miRNAs could play a role in early commitment to differentiation.

### p63-regulated miRNAs Target Several MAPKs

To understand how p63-regulated miRNAs could promote the onset of keratinocyte differentiation, we searched for their potential targets in human keratinocytes. Bioinformatics tools (TargetScan Release 5.1 and microRNA.org) were used to find hundreds of predicted targets for each miRNA.

**Figure 3:**
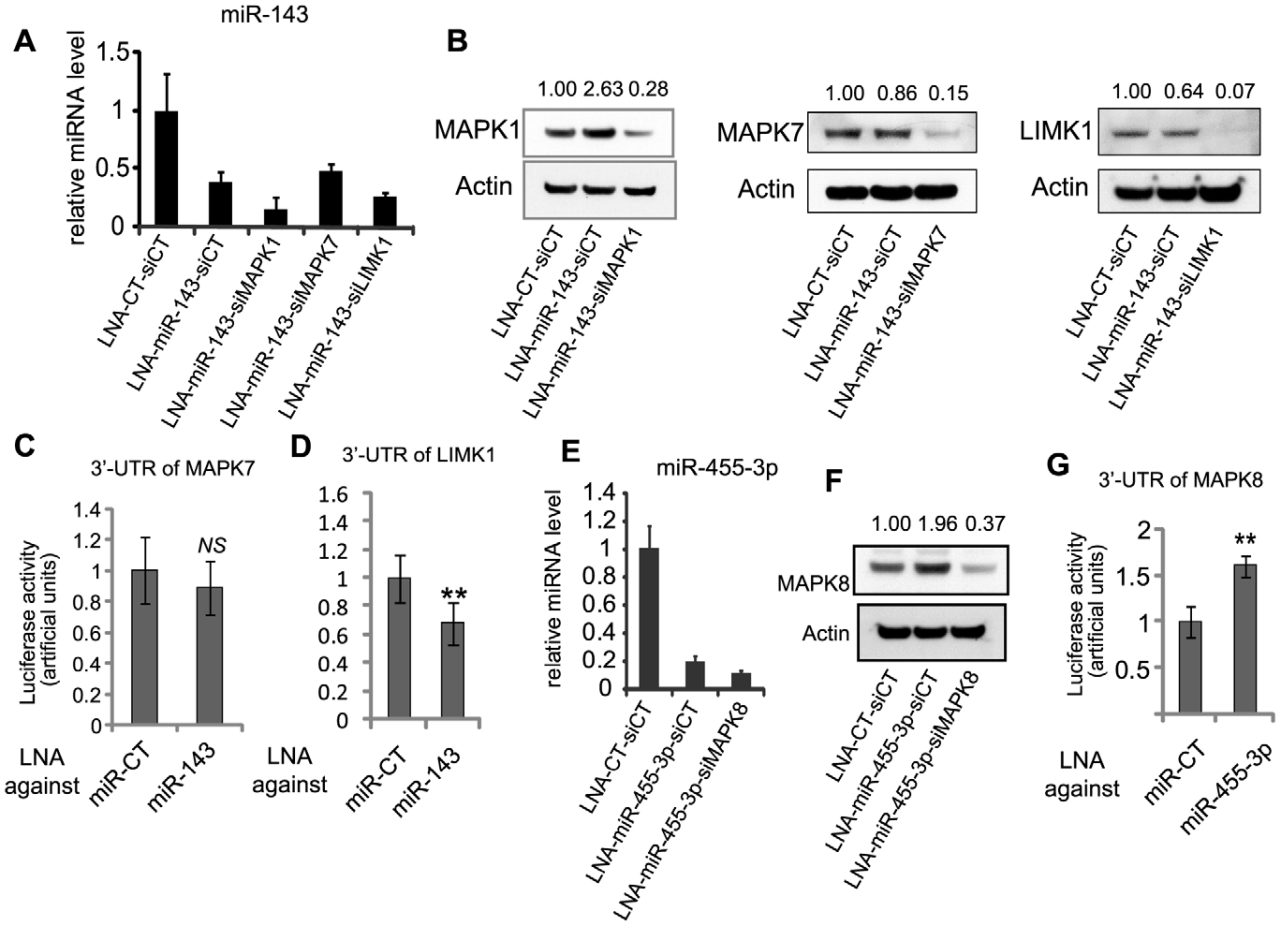
miR-143 and miR-455-3p target several MAPKs. (**A**) The effectiveness of miR-143 inhibition mediated by LNA was quantified by RT-qPCR. Error bars represent the s.d. of triplicates in all of the RT-qPCR experiments. (**B**) Expression of potential targets of miR-143 analyzed by western blotting. (**C**, **D**) The 3′UTR luciferase reporter activities of MAPK7 (**C**), and LIMK1 (**D**) were measured in HaCaT cells after specific LNA-mediated miRNA knockdown. Firefly luciferase activity was normalized to *Renilla* luciferase activity. The *t*-test was used for statistical analysis (n = 6; **, p<0.01). (**E**) The inhibition of miR-455-3p mediated by LNA was quantified by RT-qPCR. (**F**) Expression at protein level of potential targets of miR-455-3p analyzed by western blotting. All of the protein levels were quantified and normalized to β-actin. (**G**) The 3′UTR luciferase reporter activities of MAPK8 was measured in HaCaT cells after specific LNA-mediated miRNA knockdown. Firefly luciferase activity was normalized to *Renilla* luciferase activity. The *t*-test was used for statistical analysis (n = 6;**, p<0.01).

Because of the demonstrated role of MAPK signaling in keratinocyte differentiation, we have selected among the putative *in silico* targets of miR-143 several kinases belonging to the MAPK signaling cascade, such as MAPK1 (ERK2), MAPK7 (ERK5), as well as another kinase, LIMK1, not belonging to that pathway. We performed a double knockdown of miR-143 with LNA and of its predicted target with a specific siRNA in HaCaT cells. The efficiency of miR-143 silencing was systematically verified with RT-qPCR (Figure 3A). We observed on western blots that MAPK1 was significantly upregulated (2.63-fold) at protein level, upon miR-143 silencing, whereas MAPK7 and LIMK1 were not (Figure 3B). These results suggested that MAPK1 could be a direct target of miR-143, while MAPK7 and LIMK1 were probably not. Using luciferase::3′UTR reporter constructs, we demonstrated that MAPK7 and LIMK1 were not a target of miR-143 (Figure 3C & 3D). Unfortunately, we were unable to get reliable 3′UTR reporter construct for MAPK1.

Among the putative *in silico* targets of miR-455-3p, we also identified MAPKs, and we focused on this signaling cascade by selecting MAPK8. Efficient knockdown of miR-455-3p was first verified (Figure 3E) and MAPK8 was up-regulated at protein level (1.96-fold) upon miR-455-3p silencing (Figure 3F) suggesting that MAPK8 could be a direct target of miR-455-3p. We further confirmed that it was indeed the case with a luciferase::MAPK8 3′UTR reporter construct, since a LNA inhibitor of miR-455-3p increased luciferase activity (Figure 3G), while a miR-455-3p mimic strongly inhibited the reporter activity (Figure S1A).

**Figure 4:**
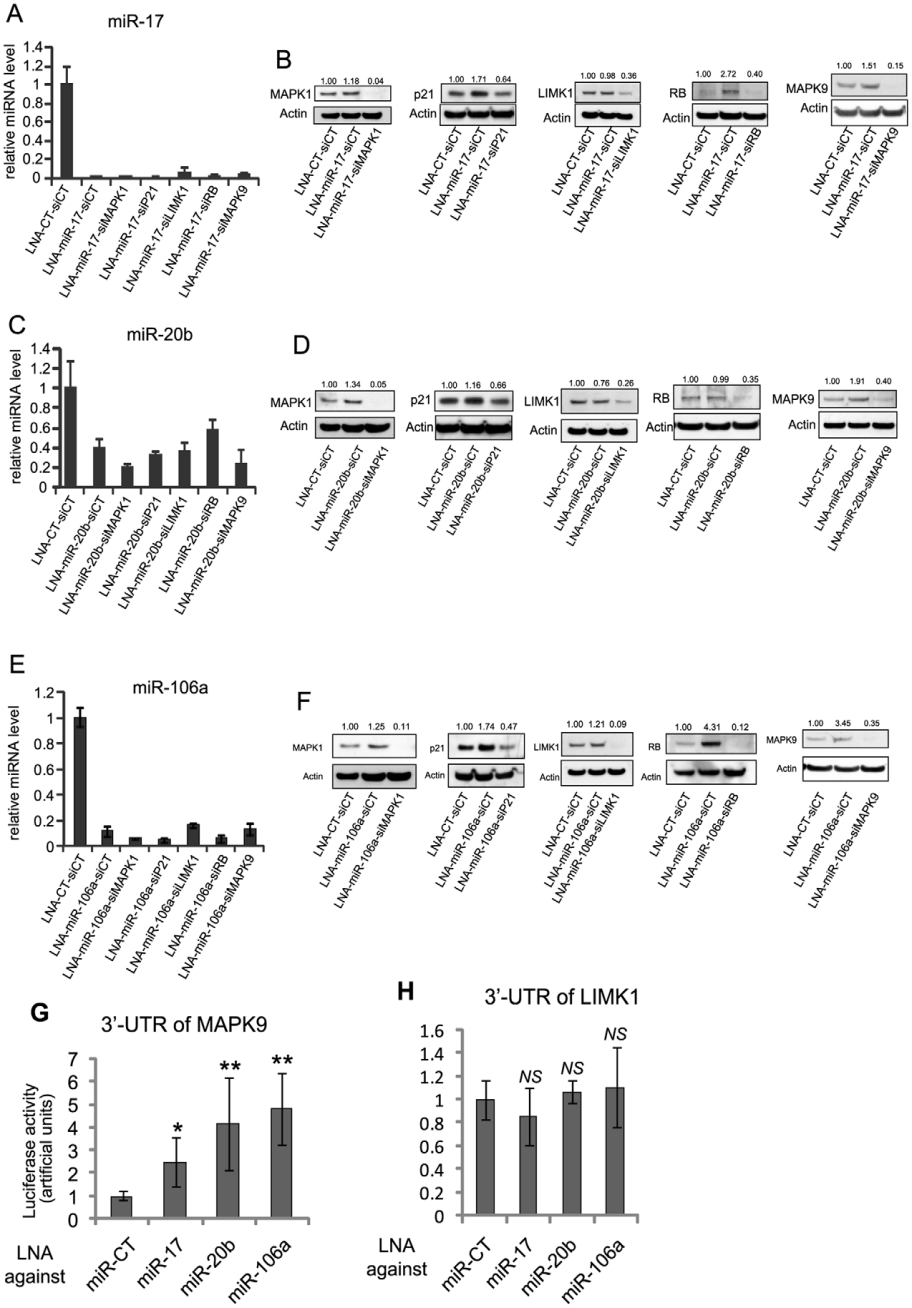
The miR-17 family also target MAPKs. (**A**, **C**, and **E**) Expression levels of miR-17 (**A**), miR-20b (**C**), and miR-106a (**E**) in double knockdown of miRNAs and of their targets was systematically measured by RT-qPCR. Error bars represent the s.d. of triplicates in all of the RT-qPCR data. (**B**, **D**, and **F**) Expression at protein level of potential targets of miR-17 (**B**), miR-20b (**D**), and miR-106a (**F**) were assayed by immunoblotting. All of the protein levels were quantified and normalized to β-actin. (**G**, **H**) The 3′UTR luciferase reporter activities of MAPK9 (**G**), and LIMK1 (**H**) were measured in HaCaT cells after specific LNA-mediated miRNA knockdown. Firefly luciferase activity was normalized to *Renilla* luciferase activity. The *t*-test was used for statistical analysis (n = 6; *, p<0.05; **, p<0.01).

We found that miR-17, miR-20b and miR-106a were strongly downregulated in keratinocytes lacking p63. MiR-17, miR-20b and miR-106a belong to the miR-17 family. Using bioinformatics tools, we obtained a list of putative targets (approximately 1,000 genes) of the miR-17 family. In agreement with our previous results, we focused on MAPK1, MAPK9, LIMK1, but also analyzed RB and p21 for their role in cell cycle regulation. While we systematically confirmed that miR-17 was inhibited (Figure 4A), western blots demonstrated that MAPK9, RB, and p21 were up-regulated upon miR-17 depletion, suggesting that they could be a direct target of miR-17, while MAPK1 was only slightly increased upon inhibition of miR-17 and LIMK1 was not (Figure 4B). MAPK1, MAPK9 and p21 were up-regulated upon inhibition of miR-20b as well, suggesting that they could be a direct target of this miR, but LIMK1 and RB were not (Figure 4C, 4D). Finally, MAPK1, p21, LIMK1, RB, and MAPK9 were all up regulated upon silencing of miR-106a (Figure 4E, 4F). However, MAPK1 and LIMK1 increased only faintly (Figure 4F). Using luciferase::3′UTR reporter constructs we further confirmed that MAPK9 was a direct target of miR-17, miR-20b, and miR-106a, since inhibitors of these miRNAs increased luciferase activity (Figure 4G), while mimic of the miR-17 family, on the contrary, inhibited the reporter activity (Figure S1B). We also demonstrated that LIMK1 was not a direct target of miR-17, miR-20b, or miR-106a (Figure 4H).

### MAPKs-targeting p63-regulated miRNA Induced the Onset of Keratinocyte Differentiation

We hypothesized that if one miRNA triggers differentiation through the inhibition of its target transcript, we could restore via the double knockdown of this miRNA and of its target, the expression of the early differentiation markers *K1* and *K10*, which were inhibited upon depletion of the miRNA only (Figure 2B, 2D). Preliminary experiments with a control LNA and MAPK-targeting siRNAs were performed both in HaCaT cells (Figure S2A, S2B, S2C) and primary cells (Figure S2D, S2E) to confirm efficiency of siRNA-mediated MAPKs knockdown and monitored the effect on *K1* and *K10* expression in that control conditions.

**Figure 5:**
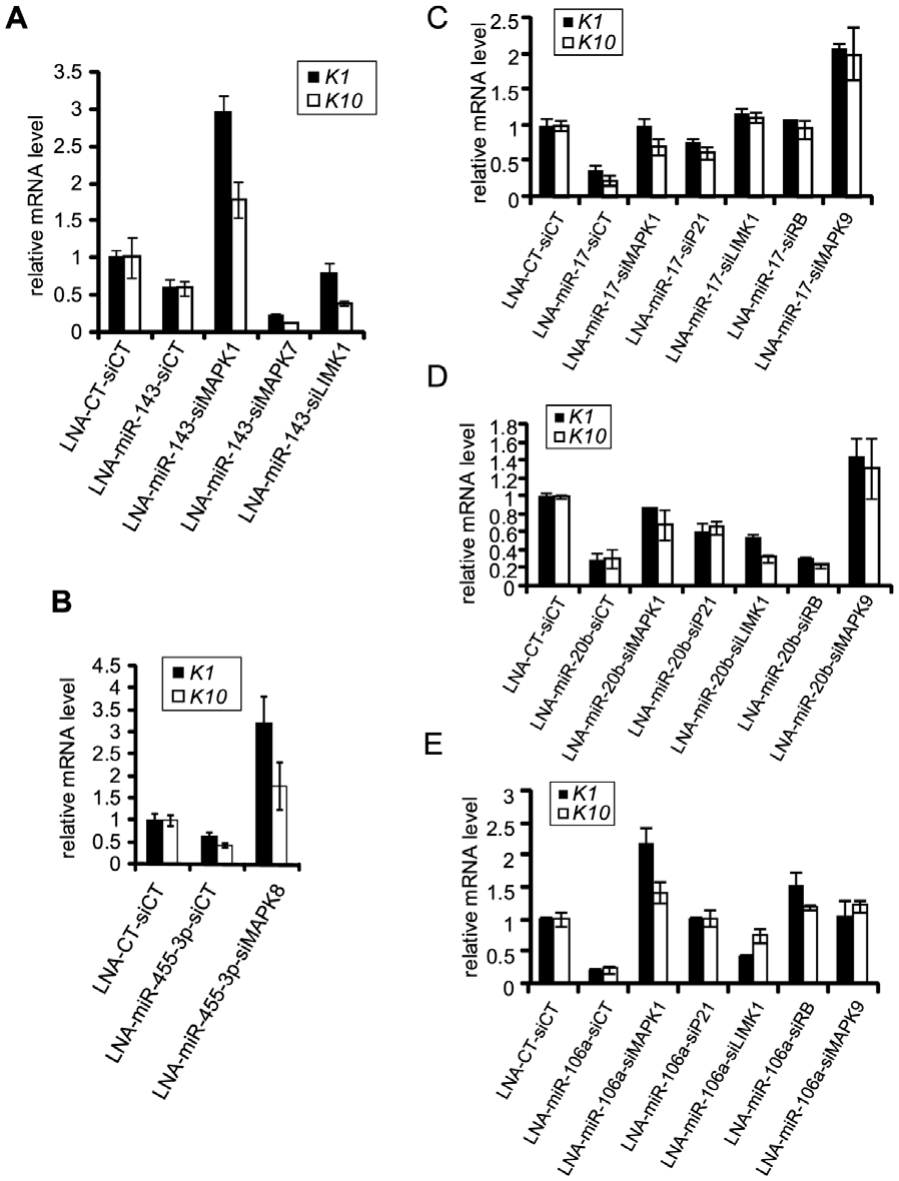
MAPKs-targeting p63-regulated miRNAs induced the onset of keratinocyte differentiation. (**A, B**) Quantification of the expression of early differentiation markers *K1* and *K10* in double knockdowns of miR-143 and of its targets (**A**) and of miR-455-3p and its targets (**B**). (**C**, **D**, and **E**) Expression of the early differentiation markers *K1* and *K10* after inhibition of miR-17 (**C**), miR-20b (**D**), or miR-106a (**E**) and their respective potential targets. The RT-qPCR experiments were done on the same RNA extracts as in figure 3A, 3E, 4A, 4C, 4E which serve as control for the inhibition of each miRNA under scrutiny. Error bars represent the s.d. of triplicates in all of the RT-qPCR experiments.

As demonstrated in figure 5A, we observed a significant up-regulation of *K1* and *K10* upon the double knockdown of both miR-143 and MAPK1, but not upon the double knockdown of miR-143 and either MAPK7 or LIMK1. RT-qPCR were done on the same RNA extracts as in figure 3A, which also serve as a control for miR-143 inhibition.

The expression of *K1* and *K10* was also rescued upon silencing of both miR-455-3p and MAPK8 (Figure 5B). Controls for miR-455-3p inhibition are in figure 3E.

We observed the up-regulation of *K1* and *K10* only when MAPK9 was silenced in miR-17-deficient cells (Figure 5C). Specific knockdown of MAPK1, p21, LIMK1, and RB showed no effect on *K1* or *K10* expression in cells lacking miR-17 (Figure 5C). MAPK1, p21, and MAPK9 silencing resulted in *K1* and *K10* up-regulation in miR-20b-depleted cells as already observed in miR-17-knockdown cells, but the inhibition of LIMK1 and RB did not have any effect on *K1* and *K10* expression (Figure 5D). Similar experiments were performed with miR-106a. The strongest up-regulation of *K1* and *K10* was observed in MAPK1 and miR-106a double knockdown cells (Figure 5E). Among other conditions only the double knockdown of miR-106a and RB led to moderate restoration of *K1* and *K10* expression (Figure 5E). Again, the RT-qPCR experiments were done on the same RNA extracts as in figures 4A, 4C, and 4E which also serve as controls for miRNAs inhibition.

It is noteworthy that RB was up-regulated upon either miR-17 (2.72 fold, Figure 4B) or miR-106a depletion (4.31 fold, Figure 4F) and we observed that the double knockdown of these genes restored the expression of *K1* and *K10*. In contrast, miR-20b depletion did not change RB protein level (Figure 4D), and we did not observe any change in the expression of *K1* or *K10* in the miR-20b/RB double knockdown.

### In vitro Differentiation of p63-silenced Keratinocytes was Rescued by siRNA-mediated Inhibition of MAPKs

**Figure 6:**
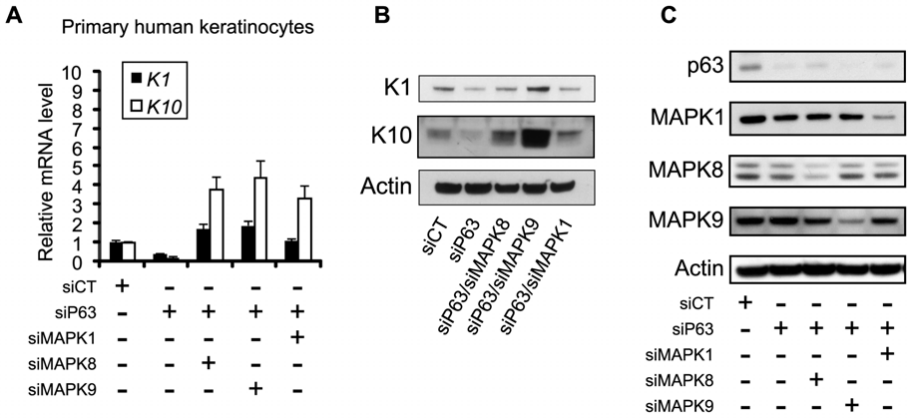
Restoration of defects in differentiation commitment in p63-deficient primary keratinocytes by the inhibition of MAPKs. (**A, B**) Primary human keratinocytes were transfected by p63 and MAPK-specific siRNAs. Expression of the early differentiation markers K1 and K10 measured by RT-qPCR (**A**) and western blotting (**B**) at 3 days post-confluence. Error bars represent the s.d. of triplicates. (**C**) Immunoblots of p63, MAPK1 (ERK2), MAPK8 (JNK1), and MAPK9 (JNK2) in double knockdowns of p63 and MAPKs. β-actin was used as the loading control in immunoblots.

p63-knockout mice show obvious defects in epidermal development [4], [5], and loss of p63 in skin organotypic culture causes serious hypoplasia and deficiency of differentiation [6]. Based on the observations that the inhibition of certain MAPKs strongly induced the expression of *K1* and *K10* in miRNA-silenced keratinocytes, we hypothesized that we might be able to rescue differentiation commitment in p63-knockdown human primary keratinocytes by inhibiting MAPKs. Indeed, we demonstrated that the double knockdown of p63 and either MAPK8 or MAPK9 resulted in the up-regulation of both *K1* and *K10* at 3 days post-confluence (Figure 6A). The strongest restoration of *K1* and *K10* expression was observed after MAPK9 silencing (Figure 6B). The effective down-regulation of each gene was verified by western blot analysis (Figure 6C).

## Discussion

Although the importance of p63 in epidermal stratification during development [27] and in the commitment to differentiation of developmentally mature keratinocytes [6], [7] has been clearly established, the potential role of p63-regulated miRNAs in these functions remained mostly unknown. In this paper, we elucidate the role of p63-regulated, MAPK-targeting miRNAs in the onset of human keratinocyte differentiation.

Recent studies have demonstrated the involvement of miRNAs in epidermal homeostasis *in vivo* [18], [20] and keratinocyte differentiation *in vitro* [22], [28]. In particular, miR-203 has been shown to promote epidermal differentiation by directly repressing p63 [20]. The ectopic expression of miR-203 under control of the K14 promoter directly inhibits p63 expression and results in the aberrant expression of K10 in the epidermal basal layer. Therefore, miRNAs acting upstream of p63, such as miR-203, play an important role in epidermal differentiation. In this study, we have characterized multiple miRNAs, including the mir-17 family (miR-17, miR-20b, miR-106a) and miR-30a, miR-143 and miR-455-3p, which are downregulated in p63-silenced keratinocytes, suggesting that they act downstream of p63. Interestingly, the search for putative p63-binding sites in the upstream region of these miR genes in the Chip-seq database generated by Van Bokhoven’s group [29] as well as our own data [30], [31] did not reveal any obvious p63-binding sites in these regions (data not shown). Although we cannot completely eliminate direct p63-mediated transcriptional regulation, this result suggests the indirect control of the expression of these miRNAs. Because the expression of the miR-17 family is directly controlled by the proto-oncogene *MYC* [32]–[34] and because we have recently demonstrated (7) that in human keratinocytes lacking p63, *MYC* expression is down regulated, the down-regulation of miR-17, miR-20, miR-106a, miR-143 and miR-455-3p genes we observed in keratinocytes lacking p63 could be due to *MYC* down-regulation.

**Figure 7:**
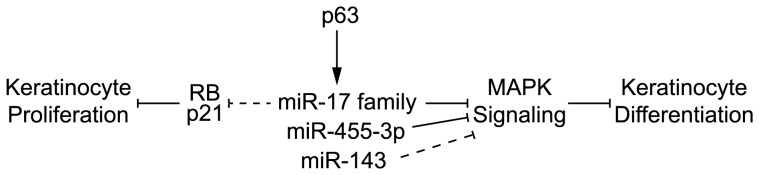
p63-regulated miRNAs participate in the control of the proliferation/differentiation balance in human keratinocytes. Under the control of p63, the mir-17 family acts on keratinocyte proliferation via down-regulation of p21, RB and keratinocyte differentiation via MAPK signaling, essentially by inhibition of MAPK9 (JNK2) which is a direct target of the miR-17 family. As we did not demonstrate in this study that p21 and RB were direct targets of miR-17, the inhibitory arrow is presented as a dot line. MiR-455-3p is also under the regulation of p63 but act only on MAPK signaling and keratinocyte differentiation through the inhibition of its direct target MAPK8 (JNK1). Mir-143 is under control of p63 and could act on keratinocyte differentiation through inhibition of MAPK1 (ERK2), but we did not demonstrate that MAPK1 was a direct target of miR-143, so the inhibitory arrow is presented as a dot line.

The tumor suppressor RB is well known for its function in cell cycle progression and epidermal keratinocyte differentiation via interaction with E2Fs [35]. RB-null mice displayed both hyperplasia and the aberrant expression of differentiation markers in the epidermis [36]. Our study demonstrated that p63-regulated miR-17 and miR-106a could target RB and p21. This result is in agreement with recent studies showing that the miR-17 family is a proto-tumorigenic group (also known as oncomir-1) [37]. As proposed in figure 7, the well-characterized role of p63 in keratinocyte proliferation could be partly modulated via the miR-17 family-dependent inhibition of RB and p21.

MiR-143 regulates smooth muscle cell plasticity [38] and prostate cell growth arrest. The miR-17 family plays a role in embryonic stem cell differentiation [39] and adipocyte differentiation [40]; however, little is known about its involvement in keratinocyte differentiation. Because p63-deficient keratinocytes fail to differentiate in skin organotypic cultures [6], we hypothesized that the p63-controlled miRNAs might be essential to trigger the early differentiation of keratinocytes. In this study, we demonstrated the involvement of p63-regulated miRNAs in the early stage of keratinocyte differentiation by monitoring the expression of K1 and K10, two well-known markers of early differentiation in the epidermis. We showed that MAPK1 (ERK2, a potential target of miR-143), MAPK8 (JNK1, a demonstrated target of miR-455-3p), MAPK9 (JNK2, a demonstrated target of miR-17, miR-20b, and miR-106a), and p21 and RB (potential targets of miR-17 and miR-106a) were strongly upregulated at protein level upon inhibition of the miRNAs. We also showed that their subsequent inhibition in miRNAs depleted cells by specific siRNAs restored differentiation commitment.

The roles of the MAPKs in cancer, apoptosis, proliferation and differentiation have been well established. JNK1(MAPK8)- and JNK2(MAPK9)-knockout mice display striking differences in epidermal proliferation and differentiation, indicating their distinct functions in epidermal homeostasis [41]. Suppression of JNK signaling promotes the differentiation of keratinocytes *in vitro* [42] and in reconstructed epidermis [43], [44]. Some of the JNK roles in proliferation and differentiation might be due to c-JUN because it has been shown to be critical in skin development [45]–[47], and JNK1 and JNK2 behave differently in c-JUN-dependent proliferation [48]. However, we cannot exclude the possibility that other JNK targets may affect differentiation. Our results clearly demonstrated that p63-controlled miRNAs targeted multiple MAPKs. Furthermore, the inhibition of MAPK signaling by siRNAs restored the delayed expression of K1 and K10 in miRNA-inhibited keratinocytes. This observation prompted us to evaluate their effects in p63-deficient cells. Strikingly, the knockdown of MAPK8 (JNK1) and MAPK9 (JNK2) could rescue early keratinocyte differentiation in p63-silenced keratinocytes.

In conclusion, our results highlight the roles of several miRNAs, particularly the miR-17 family, as regulatory intermediates for coordinating p63 with MAPK signaling in the control of the proliferation/differentiation balance in human mature keratinocytes (Figure 7).

## Materials and Methods

### Cell Culture

Primary human keratinocytes were isolated from human skin biopsies and cultured in KGM2 medium (Clonetics) in flasks coated with collagen type I (Falcon Biocoat) at 37°C and 5% CO_2_. HaCaT is a non-tumorigenic, spontaneously transformed human keratinocyte cell line [49], which was kindly provided by N. E. Fusenig (German Cancer Research Center, Heidelberg, Germany). HaCaT cells were grown at 37°C in a humidified incubator with 5% CO_2_ in Dulbecco’s modified Eagle’s medium (DMEM) containing 10% fetal bovine serum, 10^5^ units/L penicillin, 50 mg/L streptomycin and 2 mM GlutaMax (Invitrogen).

### Transient Transfection and RNA Expression Analysis

Transient siRNA and locked nucleic acid (LNA) transfections in HaCaT cells were performed with INTERFERin according to the manufacturer’s instructions (PolyPlus Transfection). Primary human keratinocytes were transfected with Nucleofector (Amaxa). Total RNA, with or without miRNAs, was extracted using RNeasy Mini Kits and miRNeasy Mini Kits, respectively (Qiagen). For mRNA quantification, 2 µg of RNA was reverse-transcribed in a total volume of 20 µl with a SuperScript II RNase H reverse transcriptase system (Invitrogen) and random primers according to the manufacturer’s instructions. Reverse transcription reactions were diluted to 500 µl of water, and 5 µl of the diluted cDNA was used for each quantitative PCR. Quantitative PCR was performed with a Platinum Quantitative PCR SuperMix-UDG Kit (Invitrogen) using the StepONE plus Real-Time PCR system (Applied Biosystems). All of the experiments were performed in triplicate, and the results were normalized to 18S rRNA expression. To evaluate miRNA expression, real-time PCR was performed using miRCURY LNA Universal RT microRNA PCR, Polyadenylation and cDNA synthesis kits (Exiqon).

All of the following siRNA oligos were synthesized by Qiagen: siRNA against all p63 isoforms (SI00055118), MAPK1 (SI00300762), MAPK7 (SI00606046), MAPK8 (SI02757209), MAPK9 (SI00300797), LIMK1 (SI00605542), p21 (SI00299810), RB1 (SI00301651), and IL1A (SI00012124) and All Stars negative-control siRNA (1027281). The following miRNA inhibitors (LNA) were obtained from Exiqon: hsa-miR-143 (138515-00), hsa-miR-455-3p (138667-00), hsa-miR-30a (138468-00), hsa-miR-17 (138461-00), hsa-miR-20b (138221-00), hsa-miR-106a (138477-00), and hsa-miR-18 (138462-00), and scramble miR (199002-04) was used as a negative control. Finally hsa-miR-455-3p *mir*Vana® mimic and hsa-miR-17 *mir*Vana® mimic were obtained from Qiagen.

### miRNA Expression Profiling

Total RNA with miRNAs was extracted using the miRNeasy Mini Kit (Qiagen). MiRNA expression profiling was performed with a miRCURY LNA microRNA Array platform at Exiqon, and the manufacturer analyzed the raw data to obtain miRNA expression results. The validation of microarray results was confirmed with qPCR at Exiqon. Three biological replicates (independent HaCaT cell transfection and independent RNA extraction) were performed for the microarrays. We considered the miRNAs with p<0.001 to be statistically significant (two-tailed t-test) and used these for further studies.

### Immunoblotting

Total cellular protein was extracted using RIPA buffer (50 mM Tris-HCl, pH 7.4, 1% Nonidet P-40, 0.25% sodium deoxycholate, 150 mM NaCl, 1 mM EDTA, 1 mM NaVO_4_, and 1 mM NaF) supplemented with Protease Inhibitor Cocktail (Roche Applied Science) and 1 mM sodium orthovanadate. After quantification with a BCA protein assay kit (Pierce), 20 µg of protein from each sample was separated with a NuPAGE Novex 4–12% Bis-Tris gel (Invitrogen) and then transferred to a PVDF membrane (GE Amersham). The membranes were blocked for 1 hour at room temperature in TBS containing 0.1% Tween-20 and 5% nonfat dry milk. The membranes were then incubated overnight at 4°C in 1% milk/TBST with primary antibody followed by 1 hour at room temperature with HRP-conjugated secondary antibody. Detection was performed with an ECL Plus kit (Amersham). The primary antibodies used in this study included antibodies against TP63 (4A4, sc-8431), MAPK8 (sc-1648), MAPK9 (sc-827), and p21 (sc-397) from Santa Cruz Biotechnology, Inc.; MAPK1 (4696), MAPK7 (3372), and LIMK1 (3842) from Cell Signaling Technology; RB1 (554136) from BD Pharmingen; human cytokeratin 1 (PRB-149P) from Covance; and β-actin (A3854) from Sigma.

### 3′-UTR Luciferase Reporter Assay

All of the 3′-UTR constructs were obtained from SwitchGEAR Genomics. HaCaT cells were cotransfected with luciferase reporter constructs and LNA by nucleofection (Amaxa). A TK-*Renilla* reporter was used as an internal normalization control. At 48 hours post-transfection, luciferase activity was measured using a Dual-Luciferase Reporter Assay System (Promega), and relative luciferase activities were normalized to *Renilla* luciferase activity.

## Supporting Information

Figure S1 **The 3′-UTR luciferase reporter activities of MAPK8 (A), and MAPK9 (B) were measured in HaCaT cells in presence of 20 nM final of miR-455-3p or miR-17 family mimics.** NT corresponds to non transfected cells along with luciferase activity buffer. Firefly luciferase activity was normalized to *Renilla* luciferase activity. The *t*-test was used for statistical analysis (n = 6; **, p<0.01).
(TIF)

Figure S2 **Effect of MAPKs silencing on keratinocytes differentiation.** The efficiency of MAPK1, MAPK8 and MAPK9 knockdown in presence of a control LNA was verified by RT-qPCR in HaCaT cells (**A**) or in human primary keratinocytes (**D**). The expression of early differentiation markers *K1* and *K10* was quantified by RT-qPCR 72 h after the double transfection of control LNA (20 nM) and MAPKS-targeting siRNA (20 nM) (**B**) or 96 h after transfection (**C**) in HaCaT cells. (**E**) Expression of *K1* and *K10*, 72 h after double transfection in human primary keratinocytes. Error bars represent the s.d. of triplicates in all of the RT-qPCR experiments.
(TIF)

Table S1 **List of differentially expressed miRNAs in p63-knockdown HaCaT cells compared with control siRNA-treated cells.** For each miRNA p-value and fold change are given. Each fold change is the average of three independent biological replicates (independent siRNA transfection and independent extraction of RNA).
(XLS)
