# Supplementary figures and images for: The miR-17 Family Links p63 Protein to MAPK Signaling to Promote the Onset of Human Keratinocyte Differentiation
Source: PLoS One. 2012 Sep 24;7(9):e45761. doi: 10.1371/journal.pone.0045761 (PMC3454365; doi:10.1371/journal.pone.0045761)

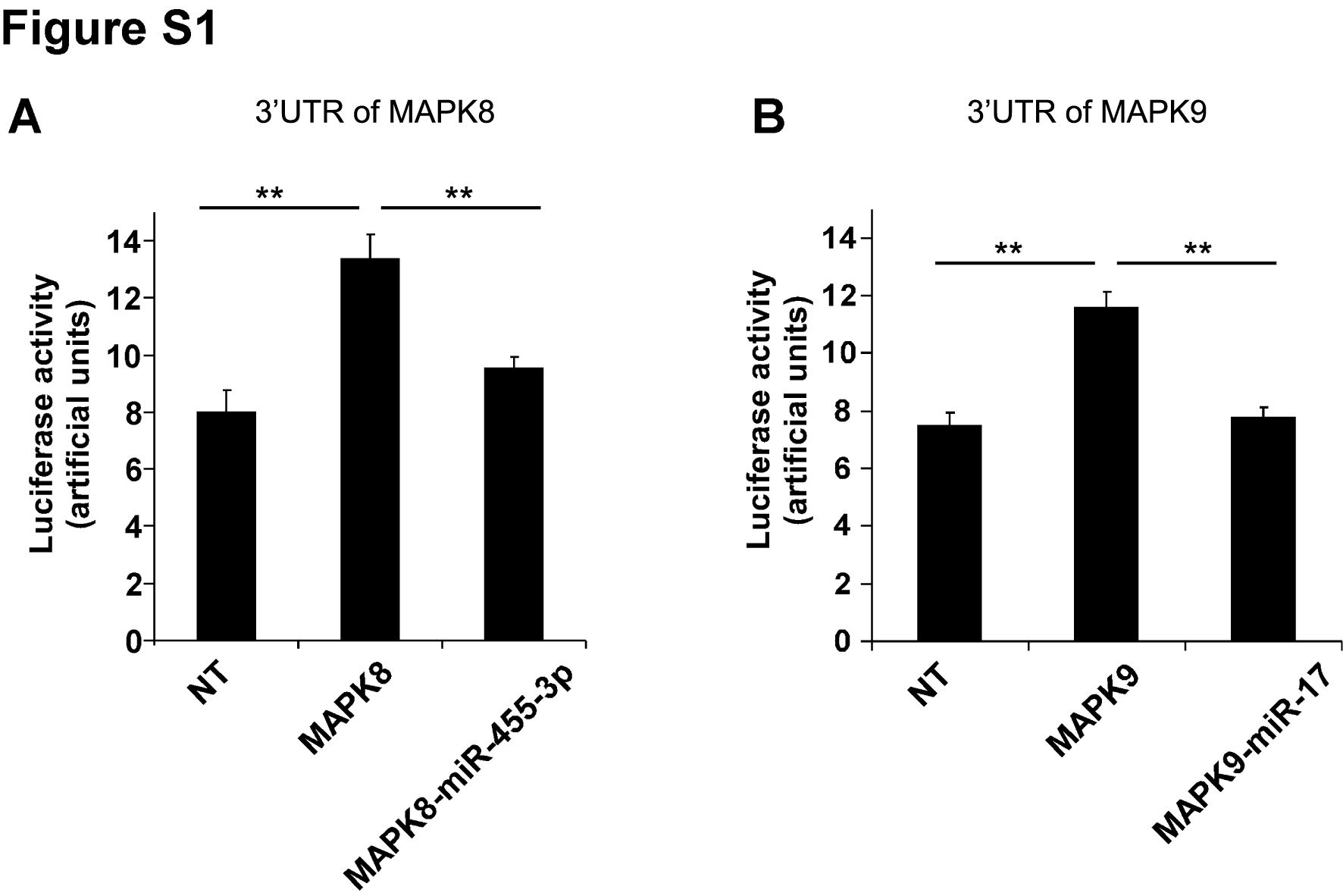

Supplement: Figure S1 — The 3′-UTR luciferase reporter activities of MAPK8 (A), and MAPK9 (B) were measured in HaCaT cells in presence of 20 nM final of miR-455-3p or miR-17 family mimics. NT corresponds to non transfected cells along with luciferase activity buffer. Firefly luciferase activity was normalized to Renilla luciferase activity. The t-test was used for statistical analysis (n = 6; **, p<0.01). (TIF) [file pone.0045761.s001.tif]

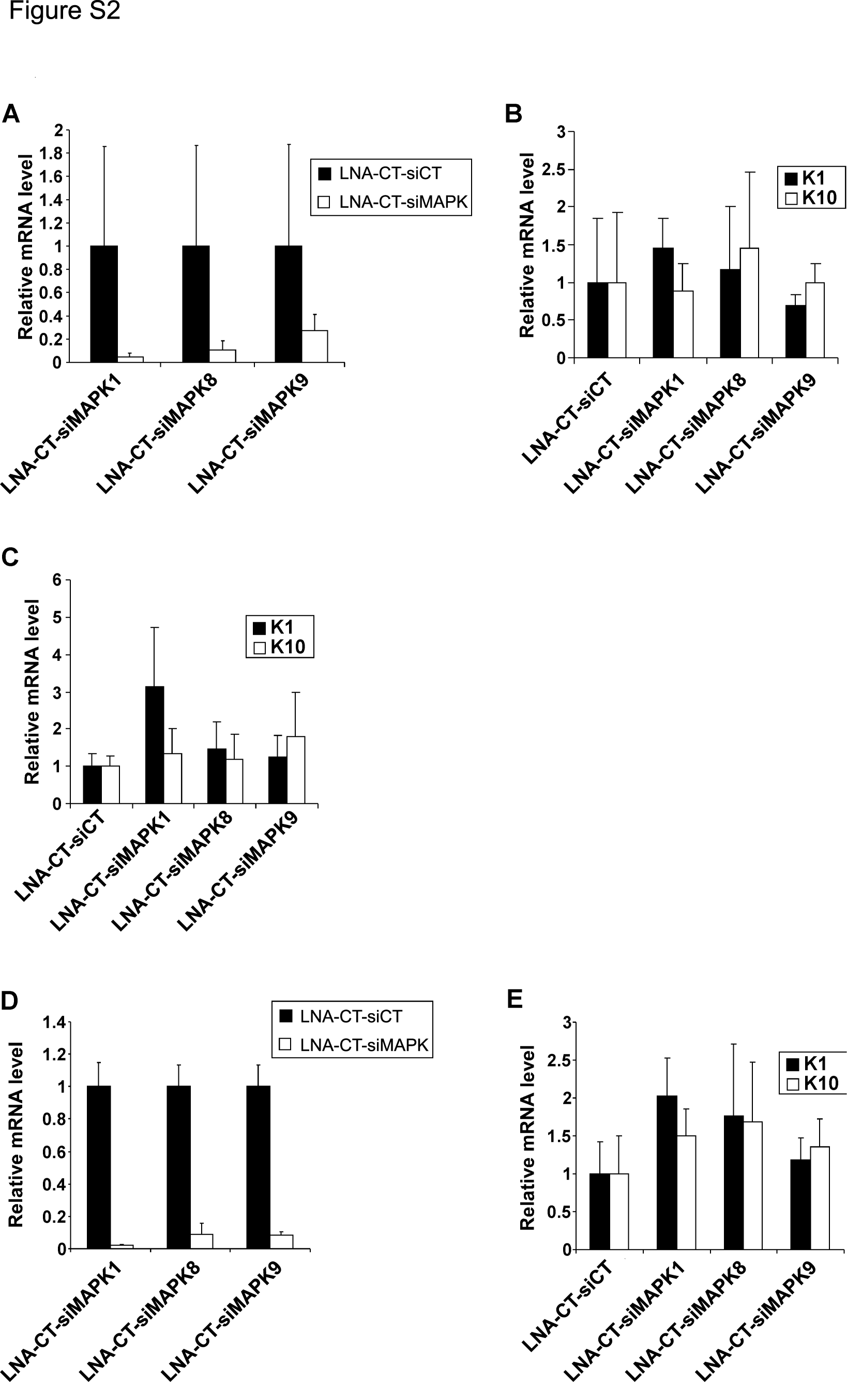

Supplement: Figure S2 — Effect of MAPKs silencing on keratinocytes differentiation. The efficiency of MAPK1, MAPK8 and MAPK9 knockdown in presence of a control LNA was verified by RT-qPCR in HaCaT cells (A) or in human primary keratinocytes (D). The expression of early differentiation markers K1 and K10 was quantified by RT-qPCR 72 h after the double transfection of control LNA (20 nM) and MAPKS-targeting siRNA (20 nM) (B) or 96 h after transfection (C) in HaCaT cells. (E) Expression of K1 and K10, 72 h after double transfection in human primary keratinocytes. Error bars represent the s.d. of triplicates in all of the RT-qPCR experiments. (TIF) [file pone.0045761.s002.tif]
